# Understanding Risky Behavior: The Influence of Cognitive, Emotional and Hormonal Factors on Decision-Making under Risk

**DOI:** 10.3389/fpsyg.2017.00102

**Published:** 2017-02-01

**Authors:** Petko Kusev, Harry Purser, Renata Heilman, Alex J. Cooke, Paul Van Schaik, Victoria Baranova, Rose Martin, Peter Ayton

**Affiliations:** ^1^Department of Psychology, Kingston University LondonLondon, UK; ^2^Department of Psychology, Nottingham Trent UniversityNottingham, UK; ^3^Department of Psychology, Babeş-Bolyai UniversityCluj-Napoca, Romania; ^4^Department of Psychology, Teesside UniversityMiddlesbrough, UK; ^5^Department of Psychology, Lomonosov Moscow State UniversityMoscow, Russia; ^6^Department of Psychology, City University of LondonLondon, UK

**Keywords:** decision-making, risky behavior, risky context, risky content, cognitive factors, emotional and hormonal factors

## Abstract

Financial risky decisions and evaluations pervade many human everyday activities. Scientific research in such decision-making typically explores the influence of socio-economic and cognitive factors on financial behavior. However, very little research has explored the holistic influence of contextual, emotional, and hormonal factors on preferences for risk in insurance and investment behaviors. Accordingly, the goal of this review article is to address the complexity of individual risky behavior and its underlying psychological factors, as well as to critically examine current regulations on financial behavior.

## How Do Cognitions and Emotions Influence Risky Behavior

Theories of judgements and decision-making have explored the influence of economic and psychological factors on risky behavior. However, very little research has offered a holistic exploration of the influence of contextual, content, emotional, and hormonal factors on preferences for risk in insurance and investment behaviors. Moreover, in this review article, we aimed to address this gap: we offered evidence that these factors have an independent influence on preference formation and behavior under risk. Accordingly, our review highlights a need to investigate how variation in these factors produces variation in decision-making under risk.

### Utilitarian Expectations and Decision Norms

Non-psychological (e.g., economic) norms, rules and regulations are the expected decision environment of any human financial behavior. Thus, human decision agents are (i) expected to act ‘normatively’ and make rational decisions, and (ii) evaluate and assess according to normative criteria and utilitarian expectations. These expectations are quite often unrealistic, as human agency (psychological processing and behavior) is assessed and measured with externally designed economic tools (e.g., rational norms and rules), which typically do not include psychological parameters (e.g., Von Neumann and Morgenstern, 1947). In this article, we summarize theoretical and empirical evidence for the role of several major psychological and biological factors influencing human agency, expected normative acts and respective behavioral outcomes.

According to normative (economics) and descriptive (psychology) theories of decision-making (e.g., Von Neumann and Morgenstern, 1947; Tversky and Kahneman, 1992) human decision-making behavior is informed by computational integrations of attributes such as probabilities and money (in a decision-making context). Furthermore, human agents engaging in financial behaviors (e.g., investment and protective) are supposed to obey normative expectations by performing utility trade-offs between the computed outputs (psycho-economic variables such as expected values) and certain monetary alternatives (see Kahneman and Tversky, 1979; Tversky and Kahneman, 1992). A rational decision-maker must choose the alternative with the highest utility, independent of decision-making context (i.e., verbal presentation and description). Crucially, normative theory implies that any information not pertinent to calculating the expected values of the options should not influence the choices.

### Risky Behavior: The Role of Decision Context, Content, and Experience

In many studies measuring human risky preferences the respondents are typically invited to make a decision, which involves a choice between a probabilistic/uncertain option (such as 50% chance of winning £4000) and a sure option (certain win of £2000). For example, based on tasks with hypothetical monetary gambles, prospect theory (Tversky and Kahneman, 1992) predicts that two psychological parameters (contextual loss and gain framing and probability levels employed in the gamble) underlie human risky decisions. However, recent research (Kusev et al., 2009, 2012, 2016; Vlaev et al., 2010; Kusev and van Schaik, 2011) explored the role of memory and decision-making content on risky decisions. These experiments (Kusev et al., 2009) demonstrated that the behavioral effects of decision-making content (type of utility and associated memory) are independent of that of context (e.g., textual summaries and descriptions). Specifically, differences in contextual descriptions of risk (e.g., probability levels), monetary amounts, as well as accessibility to decision-making content (e.g., vividness of events in memory and activated emotions) alter respondents’ risk preferences and choices (e.g., Slovic, 1987; Kusev et al., 2009; see also Tversky and Kahneman, 1992; Tversky and Wakker, 1995). Furthermore, theorists have argued that the particular combination of contextual factors, accessibility to content of decision-making utility, task type and demands, and computational skills trigger a particular type of behavioral response – normatively rational or irrational risky behavior (e.g., Kusev and van Schaik, 2011).

It is also well established that human agents experience decision-making information rather than relying on descriptions of decision-making information. For example, risky events in the real world are experienced sequentially and often without contextual summaries (Hertwig et al., 2004; Stewart et al., 2006; Kusev et al., in press). However, some risky events are not experienced individually over time, but are reviewed retrospectively and can also immediately be inspected holistically as with learning about decisions from descriptions (Tversky and Kahneman, 1992). Nevertheless, with both decision-making experiences agents refer to exactly the same utilitarian data before expressing their risk preferences. For example, research in insurance decision-making (Kusev et al., 2009) revealed that both decisions from experience and descriptions have an independent influence on risk preference. People’s experiences of events leak into decisions even when normative risk information is explicitly provided (Kusev et al., 2009). Specifically, respondents exaggerated the risk for accessible utilitarian events in memory, indicating that variation in decision content produces variation in preferences for risk (Kusev et al., 2009; Kusev and van Schaik, 2011).

Insurance decisions and other precautionary behaviors are aimed at minimizing or avoiding risk. Typically the benefits of taking precautionary actions exemplify risk-averse behavior (Hershey and Schoemaker, 1980; Kusev et al., 2009). Health-related insurance behavior and private long-term care insurance are of particular interest for policy makers and the general public. Results from a recent survey on retirement-planning behavior revealed that emotions might be important predictors of long-term care insurance purchase intentions (Tennyson and Kyung Yang, 2014). More specifically, it was found that respondents for whom family is a greater source of life satisfaction express higher purchase intentions. The authors suggest that strong emotional ties with the family may lead to a stronger desire to prevent burdening them with long-term care responsibilities (Tennyson and Kyung Yang, 2014). Respondents’ willingness and decisions to buy insurance are not influenced only by cognitive or economic factors, but also emotional factors come into play and in the following section we provide empirical arguments to support this claim.

### Emotions and Emotion Regulation Strategies in Risky Behavior

In this section, we will focus on the effects of emotions on decision-making. Namely, some of the most influential theoretical and empirical perspectives on the complex interactions between affective processes and decision processes will be reviewed, followed by applications in insurance and investment decisions. In the last part of this section, we will introduce the concept of emotion regulation (ER) and present recent studies looking into the effects of the mechanisms people use to control emotions on their decision performance.

While mainstream economics tends to employ axiomatic normative assumptions about decision agency, research evidence from psychology and neuroscience provide strong empirical support for the effects of human emotions on risky behavior (e.g., BR25; BR9; BR29; BR35; BR37). The main efforts in the studies of emotion-decision interaction are in understanding the role of emotions in decision-making (Loewenstein and Lerner, 2003; Damasio, 2005), and their neural underpinnings (Panksepp, 1998).

Several models and theories on the emotion-decision processes interplay attempt to explain and predict the decision consequences of affective factors. Loewenstein and Lerner (2003) present a systematization of possible interactions between emotions and decision-making. One important theoretical contribution suggested by these authors is the classification of emotions as *immediate* or *anticipated* emotions. Anticipated emotions refer to the emotions people expect to feel as a consequence of choosing one decision alternative over another. Expected utility theory or other consequentialist models assume that people predict utilitarian and emotional consequences (associated with choice options), and choose the option that maximizes positive emotions and minimizes negative emotions. In contrast, immediate emotions include all affective states that the decision-maker has at the time of the decision. These immediate emotions can be directly related to the decision context. For example, when a person experiences anxiety in front of a risky situation, or indirectly, when the decision-maker feels happy, and more optimistic about positive outcomes of a risky decision (Loewenstein and Lerner, 2003). Notably, in the last few decades, economists have chiefly been preoccupied with studying anticipated emotions, such as regret and disappointment (Loomes and Sugden, 1982), whereas psychologists have focused on immediate emotions (Loewenstein, 2000).

Since the publication of Loewenstein and Lerner’s (2003) work, numerous studies have explored the effects of anticipated and immediate emotions on a large variety of decision tasks and contexts. Examples are in behavioral risk-taking and perception of risk (e.g., Slovic et al., 2004; Damasio, 2005; Miu et al., 2008), judgements of happiness and overall satisfaction with life (Schwarz and Clore, 1983), consumer decision-making and the endowment effect (Lerner et al., 2004; Han et al., 2007), as well as implications of social mood and individual differences in stock market indexes and other financial outcomes (Olson, 2006). Accordingly, there has been ongoing debate about which emotion category has more influence on risky behavior. For instance, Schlösser et al. (2013), offered strong empirical support for the claim that immediate emotions predict risky decisions, beyond the effects of anticipated emotions or the subjective probability attached to outcomes. In a similar vein, Grable and Roszkowski (2008) showed that decision-makers in a happy mood have higher levels of financial risk tolerance, holding bio-psychosocial and environmental factors constant. Using a mood induction procedure, Stanton et al. (2014) reported that a happy mood induction increased risk-seeking behavior compared to neutral mood, whereas a sad mood induction procedure did not induce behavioral differences in comparison to neutral mood.

However, the existing literature provides a mixed image regarding the effects of positive and negative moods or affective states. For instance, positive emotional states are associated with increased problem solving capacity (Isen, 1984, 1987, 1993), remembering positive events (Bower, 1981), increased risk-taking (Kahn and Isen, 1993), and optimism toward the possibility of living positive events in the future (Wright and Bower, 1992; Nygren et al., 1996). Other studies have linked positive mood or emotional states to reduced risk-taking. For instance, Isen and Patrick (1983) put forward the “*mood maintenance theory*” which states that people in happy moods are more reluctant to take risks since they don’t want to undermine their positive emotional state (Isen et al., 1988).

On the other hand, negative affective states were shown to predispose individuals toward remembering negative past events (Bower, 1981) as well as overestimating the chances of a negative event in the future (Johnson and Tversky, 1983). Consensus in the scientific literature regarding the effects of negative emotions on risk-taking is also lacking. Whereas some papers documented the fact that experimentally induced negative emotions as well as anxious and depressive states lead to more risk-averse preferences (Leith and Baumeister, 1996; Yuen and Lee, 2003; Miu et al., 2008; Heilman et al., 2010), other authors provide empirical evidence supporting a positive relation between negative emotions and risk-taking (Mittal and Ross, 1998; Raghunathan and Pham, 1999; Bruyneel et al., 2009).

As reviewed in the preceding paragraphs, most theories and empirical studies of affective influences on decisional processes have contrasted the effects of positive emotions on decision-making with the effects of negative emotions. These studies started from the assumption that all positive emotions and all negative emotions, respectively, should have similar impact on decision-making in general, or risk-taking, in particular. However, as previously reviewed, behavioral data regarding affective influences on risk-taking are less consistent. Lerner and Keltner (2000, 2001) proposed the “*appraisal tendency hypothesis*” to address this issue. Their theory asserts that each emotion is associated with a specific appraisal dimension, which, in turn, will determine the influence of specific emotions on judgements and decisions. For instance, based on Lerner and Keltner’s (2000, 2001) results, induced fear was associated with pessimistic judgements of future events and risk-averse choices, whereas induced anger was associated with more optimistic judgements and a more risk-seeking behavioral pattern. In conclusion, appraisals of certainty and control moderate and/or mediate emotional effects on risk-taking. Taking appraisal dimensions of same-valence emotions into account might eliminate some of the inconsistency in previously reported results.

Looking specifically at financial decision-making, there are several studies that unravel the complex dynamics between emotions and risky decisions. Van Winden et al. (2011) observed that the timing of the resolution of risk (the time that passes between the risky decision and the consequences of that decision) and anticipatory emotions predicted investment behavior. Delaying the resolution, for negative anticipatory emotions (with high-probability success) discouraged investment behavior, whereas delaying the resolution for positive anticipatory emotions (with low-probability success) encouraged the investment behavior. Similarly, Gambetti and Giusberti (2012) argued that trait anxiety predicts low-risk investment decisions, whereas trait anger is associated with higher financial risk-taking. In addition, experimental findings (Guven, 2012) revealed that happier people spend less, and are (i) less likely to have debts, (ii) more concerned about the future and thus save more money. Likewise, consumer behavior is also influenced by affective states: a study conducted by Cryder et al. (2008) showed that people tend to spend more money when they are in a sad mood.

With regard to precautionary behavior, experimental results have shown that buying insurance is more attractive for items with affective descriptions inducing participants’ fear of losing the items (Petrova et al., 2014). Accordingly, a survey study, analyzing data from UK households who either had experienced flooding or who were at risk of flooding, found that people’s protective decisions were influenced more by anticipated negative emotions, such as anxiety or insecurity, than by material and financial considerations (Harries, 2012). If the decision to insure against flooding is partly determined by affective reactions and experience, more research should be carried on in this area, in order to inform better public policy.

However, there is evidence that people employ regulation strategies designed to alter their emotional reactions. Under optimal circumstances, the success of regulatory strategy assures a good emotional and social functioning of the individual. Nevertheless, when ER mechanisms are malfunctioning, they can increase the risk for developing symptoms of psychiatric disorders (Davidson et al., 2000; Phillips, 2003). One of the most influential approaches in the study of emotion and ER is the process model of emotions (Gross, 1998, 2002). ER is a construct that subsumes all the actions that people take in order to control which emotions they have, when they have them and how they experience or express those emotions (Gross, 2002).

One ER strategy that has received particular attention is cognitive reappraisal (Gross, 2002; Ochsner and Gross, 2007; Siemer et al., 2007). This strategy implies that changing the situation’s meaning alters its emotional impact. In contrast, the ER strategy of expressive suppression involves inhibiting ongoing emotion-expressive behaviors (Gross, 1998; Ochsner and Gross, 2007). Individual differences in habitual use of ER strategies (i.e., cognitive reappraisal and expressive suppression) have been associated with effects on affective, social, and cognitive functioning of the individual, physiological changes or even psychological well-being (John and Gross, 2004).

Because of the effectiveness of ER strategies, it is possible that these strategies might, in fact, moderate the involvement of emotions in financial decision-making. Most of the previous studies on emotion and decision-making have not controlled for ER. Therefore, the effects of ER on financial decision-making, ranging from ‘coloring’ the content of thoughts to interfering with information processing and context (previously attributed to acute emotions) might actually be moderated by ER strategies such as cognitive reappraisal or expressive suppression. Only recently, however, have scholars begun to investigate these possibilities, and in doing so they have discovered that ER can indeed moderate the effects that task and incidental emotions have on decisions (Heilman, 2006, 2014; Kahneman and Frederick, 2007; Heilman et al., 2010; Miu and Crisan, 2011; Heilman and Miclea, 2015; Szasz et al., 2016).

Psychologists and economists investigating risky behavior should be interested in understanding how and in what contexts emotions influence risky attitudes and behavior. Moreover, how can these emotions be efficiently controlled, in order to reach the utilitarian beneficial decision outcomes (e.g., the option with the highest utility overall)? Although much has been written about neurotransmitters (e.g., dopamine, Norbury and Husain, 2015) in relation to risk, a critical area that, until recently, has been relatively neglected by psychologists and economists is the hormonal underpinning of risk. Hormones are particularly relevant to understanding context-specificity, given that some of their blood levels can vary according to context and some of their effects are transient and short-lasting (e.g., Resko and Eik-Nes, 1966; Ditzen et al., 2009). The following section gives an overview of the hormonal predictors of risk-taking.

## Hormonal Predictors of Risk-Taking

### Hormonal Correlates of Changes in Environment

Cortisol is known as the stress hormone, with higher levels reflecting increased amounts of stress and lower psychological well-being (e.g., Dickerson and Kemeny, 2004). Dickerson and Kemeny (2004) highlighted that the relationships between a number of putative psychological stressors and cortisol levels have not been consistently established in the literature. The hypothalamic–pituitary–adrenocortical (HPA) network is triggered by the release of corticotrophin-releasing hormone, in turn stimulating release of adrenocorticotropin hormone from the anterior pituitary, finally causing secretion of cortisol into the bloodstream from the adrenal cortex (e.g., Sapolsky et al., 2000). The activation of the HPA network has been associated with a range of psychological changes, most notably stress (e.g., Selye, 1956).

A common formulation is that the adrenocortical system is activated by circumstances in which the individual is prevented from pursuing goals, or where those goals are otherwise threatened (Carver and Scheier, 1999). Within this framework, in which the individual’s motivation is central, stress is triggered because of the adaptive relevance of interference with goals. Basic threats to one’s survival or safety elicit release of cortisol from the HPA network, serving an adaptive purpose, given that cortisol augments the bioavailability of energy for fight or flight, amongst other associated physiological optimisations for survival-relevant behaviors (Lovallo and Thomas, 2000). Although such self-preservatory threats are generally considered the archetypal conditions that stimulate cortisol release (Sapolsky et al., 2000), the literature indicates that threats to other, more human-specific, goals are also associated with raised levels of cortisol.

Dickerson and Kemeny (2004) describe the ‘social self-preservation system,’ which functions to monitor the environment for threats to the individual’s social status or self-esteem. In line with this idea, the availability of social support has been associated with a reduced level of cortisol, as indexed by salivary assay (Kirschbaum et al., 1995), and a metanalysis by Dickerson and Kemeny (2004) indicated that cortisol responses were markedly augmented under conditions of social-evaluative threat. Some more recent work has broadened this research area to include the interactions of cortisol with another neurotransmitter, oxytocin, which is associated with trust and social acceptance.

Oxytocin affects the extent to which we feel trust toward others, with higher levels associated with increased trust (Zak et al., 2004; Kosfeld et al., 2005). Its release is not under deliberate conscious control, but is determined by physiological, social, and organizational conditions (e.g., Zak et al., 2004). Cortisol suppresses oxytocin, thereby directly reducing feelings of interpersonal trust, whereas oxytocin reduces cortisol levels (Ditzen et al., 2009).

Oxytocin is not just a correlate of well-being, but a direct cause of it, as shown by a number of studies, involving intranasal administration of the neuromodulator (e.g., Ditzen et al., 2009; Van IJzendoorn et al., 2012), which can demonstrably reduce the production of cortisol (Cardoso et al., 2013). For example, a study by Cardoso et al. (2013) demonstrated that intranasal administration of oxytocin attenuated cortisol level relative to placebo, although it should be noted that mood itself – the psychological instantiation of stress – was not affected by this manipulation. In another study, however, by Heinrichs et al. (2003), a combination of social support and intranasal oxytocin demonstrably lowered salivary cortisol, but also both decreased anxiety and increased calmness.

Other steroid hormones beside cortisol (estrogen and progesterone) also play an important role in human physiology throughout the life course and can be reliably measured in saliva, with careful assessment of timing in the menstrual cycle (Gavrilova and Lindau, 2009). Progesterone, estrogen, and the adrenal-stress hormones (such as cortisol) are intimately linked (Edwards and Mills, 2008). Stress knocks the hormonal patterns out of rhythm and also places a greater demand on the body’s nutrient reserves, leading to tiredness and a vicious circle of feeling less able to cope with stress. Estrogen has been shown to increase non-reproductive behaviours in mice, including anxiety, fear, and physical activity level (running; Morgan and Pfaff, 2001). Progesterone plays an important role in brain function and is often called the “feel-good hormone” because of its mood enhancing and antidepressant effects (Wierman, 2007).

### Hormonal Response to Challenges, Risk, and Ambiguity

Cortisol has recently been implicated in accounts of fluctuations in risk-taking behavior. Coates and Herbert (2008) investigated the impact of market volatility – entailing a lack of informational availability, or ambiguity – on a population of traders in the City of London, finding that the traders underwent a 68% increase in average cortisol levels as market volatility increased over a period of around 1 week, in addition to a markedly higher level of cortisol in the afternoon relative to those measured in the mornings which was the result of an increase in sustained effort and expected challenge. Such chronic and acute raised cortisol levels appear to have rather different effects on the cognitive system: acute increases are associated with higher motivation and more sensation-seeking (Piazza et al., 1993; Putman et al., 2010), whereas chronic raised cortisol level has been linked to impaired executive (attention) control (Liston et al., 2009), and appears to cause or exacerbate both anxiety (Korte, 2001) and depression (Sapolsky, 2000).

Kandasamy et al. (2014) hypothesized that, on the above basis, acutely raised cortisol would, if anything, increase risk-taking behavior, whereas chronic augmentation of cortisol would lead to risk aversion. In a double-blind, placebo-controlled, crossover, randomized trial involving 36 people over an 8-day period, participants’ risk preferences were measured using a series of computer tasks. Cortisol level was both acutely and chronically raised by administration of hydrocortisone, a pharmaceutical variant of cortisol. In line with the authors’ predictions, financial risk-taking was not reliably affected by acute elevation of cortisol, but was reduced by sustained elevation. Specifically, with chronic elevation of cortisol, participants opted for bets with lower variance and also lower expected returns. This finding is illustrative in calling into question the widely adopted assumption that the utility functions underlying risk preferences are unchanging through time (e.g., see Kusev et al., 2009, for a detailed questioning of such assumptions).

In addition to investigating the role of cortisol in traders, Coates and Herbert (2008) also measured level of testosterone, an androgen steroid hormone. The authors found that individuals with a higher average level of the hormone tended to generate more profit in financial transactions. Although testosterone level has been positively associated with high-risk behaviors, such as aggressive violence, irresponsible sexual behavior, and drug abuse (Middleman and Durant, 1996), little research has focused on economic risk. One of the larger and better-controlled studies was conducted by Stanton et al. (2011), involving 154 participants performing the Iowa Gambling Task, used to assess people’s willingness to take risks. In the task, participants select cards from four different decks, which differ in terms of the chances of financial rewards and penalties. Participants provided saliva samples, which were analyzed for endogenous testosterone by radioimmunoassay. The results indicated that individuals with a high level of testosterone tended to take greater risks than those people with low testosterone, with the relationship between testosterone and risk-taking similar between men and women. Although no gender differences in risk preference/aversion were found in this study, the bulk of studies have tended to find that men are less averse to risk than women.

A review by Croson and Gneezy (2009) showed that men tend to make riskier decisions in situations where risk is financial and a meta-analysis of 150 studies of gender differences in risk behavior by Byrnes et al. (1999) found that men were significantly more likely to make risky decisions in a wide variety of risk-relevant types of behavior. While endogenous testosterone is argued to augment risk-taking in a transitory manner, it has also been proposed that testosterone can have a more permanent impact on brain development *in utero* (Arnold and Breedlove, 1985). Some researchers have used the ratio of the length of the second and fourth fingers, known as the 2D:4D ratio, as a marker of the level, or at least effects, of prenatal testosterone (Manning, 2002). In contrast to the effects of plasma testosterone at any particular point in time, there is a clear causal, rather than merely correlational, interpretation of any link between 2D:4D ratio and financially risky behavior, because this ratio is fixed prior to birth (McIntyre et al., 2005) and therefore cannot be influenced by risky behavior in adulthood. Coates et al. (2009) studied the financial performance of a group of male traders and found that lower 2D:4D ratios, reflecting higher prenatal exposure to testosterone, made more money than those with higher ratios. It should be noted, however, that greater financial returns are not necessarily indicative of greater risk-taking (indeed, the opposite could be the case). Garbarino et al. (2011) investigated links between 2D:4D ratio and risk-taking directly, in a task involving financial decisions. The authors found that both men and women with lower 2D:4D ratios tended to make riskier choices. Furthermore, they argued that the 2D:4D ratio could partially account for gender differences in risk-taking. Intriguingly, there is also evidence that men’s risk-taking behavior can be modulated by the presence of women, in a way that appears to depend on prenatal exposure to testosterone: Van den Bergh and Dewitte (2006) found that men with lower 2D:4D ratios, in the presence of women, became more impulsive by selecting less desirable financial outcomes. However, the behavior of men with higher 2D:4D ratios was not altered by female presence.

Oxytocin, outlined above, has also been implicated as a modulator of risk behavior. Zak et al. (2007), using a double-blind design, administered 34 males with intranasal oxytocin and 34 males were given placebo. Participants played two games: the ultimatum game and the dictator game, in randomly formed dyads of Decision-maker 1 or Decision-maker 2. In the ultimatum game, Decision-maker 1 was allotted $10 and asked to offer a split of the money to Decision-maker 2; if Decision-maker 2 accepted the offer, the money was divided between them as per the split, but if he rejected it then no money was paid at all. In the role of Decision-maker 2, participants were asked to state the minimum acceptable offer. A generous offer was defined as one that was greater than required for acceptance. In the dictator game, the structure was similar to the ultimatum game, but Decision-maker 2 had no choice but to accept the offer by Decision-maker 1. This game was included to give a measure of altruism (by people in the role of Decision-maker 1), so that it could be dissociated from generosity in the ultimatum game. Participants given oxytocin were 80% more generous than those given placebo in the ultimatum game, but were no more altruistic in the dictator game. The authors note that this increase in generosity resembled an increase risk aversion: people in the role of Decision-maker 1 may have made more generous offers to reduce the chance of being rejected and ending up with nothing.

## Choice Preferences and Regulatory Policy

Normative models of decision-making such as expected utility theory suggest that decision-makers should make choices in order to maximize utility (Von Neumann and Morgenstern, 1947). The assumption that decision-makers follow rational normative assumptions, however, has been refuted frequently in the literature (Tversky and Kahneman, 1992). Decision-makers have been shown, instead, to divert from utility maximization due to the influences from decision context, content, experience and emotions (Kahneman and Tversky, 1979; Hertwig et al., 2004; Kusev et al., 2009). Moreover, even once a choice is made, decision-makers are still vulnerable. For example, in a study employing a *sleight-of-hand* (a choice paradigm which permits the experimenter to surreptitiously manipulate the relationship between the choice and outcome that participants experience), participants shifted their political voting intentions (Hall et al., 2013). Respondents were asked to state their voting intention, and introduced to a political survey of wedge issues between two political coalitions. Using sleight-of-hand, voting intention replies were altered and placed in the opposite political coalition and participants were invited to reason about the manipulated wedge issues. Remarkably, participants detected no more than 22% of the manipulated replies.

The need to protect decision-makers by legislation is evident. Due to the vulnerability and fragility of choice preferences, it is essential that consumers are protected by legislation not only from explicit deception but also from decision subtleties, context, and content. In this section, we shall briefly explore and evaluate aspects of European financial legislation, directives, and communications regarding both consumer protection and financial education, in relation to decision-making theory.

The Consumer Credit Directive (European Parliament, 2008) sets out to standardize information given regarding consumer credits. One normative aspect in this document is about advertising regulations regarding credit products. They must contain information about the interest rate, credit amount, and the annual percentage rate (APR). Whilst APR incorporates additional costs rather than just representing the borrowing rate, the two are closely comparable, due to the fact that they are represented and communicated in percentages. It is well established in decision-making research (e.g., Newall and Love, 2015) that consumers experience difficulties comprehending interest information, which is an issue given that credit products are typically communicated in percentages.

Similarly, the Integration of European Mortgage Credit Markets communication (European Parliament, 2007) is designed to facilitate an ‘increasing product diversity’ which is expected to ‘improve consumer confidence.’ This communication implies that more decision options are beneficial to the consumers – “…having a complete range of mortgage products…” (European Parliament, 2007). However, theorists have argued that decision-makers cannot perform multi-attribute decisions from the available options in the context (Huber et al., 1982; Simonson, 1989). Accordingly, empirical research has revealed that a choice between three decision options (e.g., three different credit products) each one spaced on two decision attributes (e.g., borrowing rate and APR) induced decision errors. For example, in their choice, consumers will always select only between two decision options on a single attribute – choosing the option with the highest utility on this attribute (and ignoring any other available attributes in the context), which is not necessarily the option with the highest utility overall. Moreover, even decision experts such as physicians are unable to overcome contextual decision biases. Redelmeier and Shafir (1995) found that additional choice options (number of medications) decreased the likelihood of prescribing medication and increased the likelihood of sending the patients to hip replacement surgery.

Both the Distance Contracts for Financial Services legislation (European Parliament, 2002a) and the EU Consumer Rights Directive (European Parliament, 2011) enforce consumers ‘Right to withdraw,’ giving the consumers 14 days opportunity window to withdraw from a financial contract. Whilst this appears to protect consumers allowing them to evaluate their needs once in possession of the item, there are, however, psychological factors at play that may bias the utility valuation of a product once owned. For example, the Endowment Effect reveals that possession of an item increases its perceived value (Kahneman et al., 1990).

The endowment effect is not only limited purely to experimental settings, but has been shown to affect trading on a grand scale within the Australian stock exchange – traders overvalued their stock portfolios (Johnstone and Furche, 2006). Whilst returning an item is possible and supported by the legislation, it is unlikely to happen as its perceived value will increase due to possession.

The Insurance Brokerage directive (European Parliament, 2002b) proposes that only financially capable should people act as insurance intermediaries. Yet, as outlined in this article, even those who make decisions professionally are susceptible to behavioral biases (Johnstone and Furche, 2006).

However, some legislations accommodate behavioral science knowledge. The EU Consumer Rights Directive (European Parliament, 2011) aims to ensure the fair treatment of consumers when purchasing goods or services. This directive bans pre-ticked boxes for additional goods or services online. Thus protecting consumers from unintended purchases of goods and services. The directive addresses the Default Effect – a tendency for decision-makers to remain with a default option, rather than switch preferences (Johnson and Goldstein, 2003).

## Summary

According to normative theory, any information that is not directly used to calculate the expected values of the decision options should not influence the choices. However, variables such as differences in contextual descriptions of risk, monetary amounts, and – importantly, as we have shown – accessibility to decision-making content (e.g., activated emotions) change risk preferences and choices (e.g., Kusev et al., 2009). There is strong empirical support for the effects of emotions on risky behavior (e.g., Guven, 2012). People are not slaves to their emotions, however, with emotional regulation strategies associated having effects on affective, social and cognitive functioning, and psychological well-being (John and Gross, 2004). There are several key hormonal correlates and determinants of risk. Cortisol, testosterone, and oxytocin have been demonstrated to be causally involved in modulating risky behavior. Chronic sustained elevation of cortisol serves to make people more risk averse, whereas both administered and naturally high testosterone increases risky behavior. Oxytocin appears to increase generosity in a manner resembling risk-aversion, but more study is needed to add confidence to this interpretation. The influence of cognitive, emotional, and hormonal factors and their interaction on decision-making have implications for regulation to improve people’s decision-making.

## Author Contributions

PK initiated the review article; PK, HP, RH, and AC drafted the manuscript; PVS, VB, RM, and PA provided feedback and suggestions; All authors approved the final version of the manuscript for submission.

## Conflict of Interest Statement

The authors declare that the research was conducted in the absence of any commercial or financial relationships that could be construed as a potential conflict of interest.
